# User-Driven Strategy for In Silico Screening of Reversed-Phase Liquid Chromatography Conditions for Known Pharmaceutical-Related Small Molecules

**DOI:** 10.3390/molecules27238306

**Published:** 2022-11-28

**Authors:** Thomas Van Laethem, Priyanka Kumari, Bruno Boulanger, Philippe Hubert, Marianne Fillet, Pierre-Yves Sacré, Cédric Hubert

**Affiliations:** 1 Laboratory for the Analysis of Medicines, University of Liège (ULiège), CIRM, 4000 Liège, Belgium; 2 Laboratory of Pharmaceutical Analytical Chemistry, University of Liège (ULiège), CIRM, 4000 Liège, Belgium; 3 Pharmalex Belgium, 1435 Mont-Saint-Guibert, Belgium

**Keywords:** reversed-phase liquid chromatography, small pharmaceutical compounds, response surface methodology, multi-criteria decision analysis, method development

## Abstract

In the pharmaceutical field, and more precisely in quality control laboratories, robust liquid chromatographic methods are needed to separate and analyze mixtures of compounds. The development of such chromatographic methods for new mixtures can result in a long and tedious process even while using the design of experiments methodology. However, developments could be accelerated with the help of in silico screening. In this work, the usefulness of a strategy combining response surface methodology (RSM) followed by multicriteria decision analysis (MCDA) applied to predictions from a quantitative structure–retention relationship (QSRR) model is demonstrated. The developed strategy shows that selecting equations for the retention time prediction models based on the pKa of the compound allows flexibility in the models. The MCDA developed is shown to help to make decisions on different criteria while being robust to the user’s decision on the weights for each criterion. This strategy is proposed for the screening phase of the method lifecycle. The strategy offers the possibility to the user to select chromatographic conditions based on multiple criteria without being too sensitive to the importance given to them. The conditions with the highest desirability are defined as the starting point for further optimization steps.

## 1. Introduction

In the analytical chemistry field, it is often essential to separate the components of a mixture to analyze them. Either to identify some components of the mixture or to quantitate them. Multiple separation modes exist to carry this out and reversed-phase liquid chromatography (RP-LC) is one of the most frequent in pharmaceutical analysis. It is also commonly used in other fields such as biochemistry, agricultural sciences, and environmental sciences. To achieve the separation of the different compounds in a mixture in RP-LC, several parameters must be defined and optimized such as stationary phase, mobile phase composition, gradient slope, gradient time, etc. Therefore, the development of an RP-LC method is generally a time-consuming and tedious process. Method development follows a minimum of two experimental phases: screening and optimization phases. The purpose of the screening phase is to acquire knowledge of the experimental space and to identify the parameters and their combinations that have a significant impact on the retention of the compounds. Only the parameters (and their combinations) that are found to be significant are kept for further optimization leading to a reduction in the number of parameters to tune during the next phase. The optimization phase takes place to reach the optimal values for each parameter (i.e., the best conditions) to achieve the separation under some constraints. In addition, robust optimization allows for ensuring the robustness of the method against small variations of the controlled parameters [1]. These two steps are usually performed with the design of experiment (DoE) methodology. Indeed, this methodology is often presented as a better alternative to one-factor-at-a-time (OFAT) designs [2]. Besides the benefit of acquiring the information in fewer experiments, the DoE methodology offers the possibility to model the interaction between the distinct parameters. The relationship between the parameters and the studied response may be analyzed with the response surface methodology (RSM). The RSM is developed to study the direction, amplitude and shape of the effect of the parameters on the response, and predict and optimize the response. It defines an equation that models the effect of the parameters on the response. Thanks to this equation, the effect of the parameters and their best combination can be inferred [1,3,4].

Response optimization can be simple when it concerns only one response but it may be complex as it is generally necessary to optimize multiple responses at the same time. In the field of chromatography, this may be interpreted as the fact that peak separation is not the only objective. Indeed, several constraints regarding the sensitivity of the detection, the analysis time, etc. are included in the decision process [5]. Different approaches were developed to achieve the best compromise between these different criteria, called multiple criteria decision analysis (MCDA). The most popular in the analytical chemistry field is desirability [1]. To apply it, an individual desirability function is defined for each criterion, to normalize it and specify if the criterion is maximized, minimized or targeted at a specific value. Eventually, it combines all those values, sometimes with different weights, in a single desirability index, with a global desirability function. Some of the advantages of this methodology are that it is easy to understand, easy to use, and versatile. It also comes with a few disadvantages such as the number of parameters to define the functions or the subjectivity of the definition of the weights [6]. Another one of its disadvantages is that the optimal solution found might be sensitive to the weights attributed to each criterion.

Generally, the development of a chromatographic method begins with some experiments defined by the DoE methodology. Modeling the retention behaviors would help reduce the number of preliminary experiments realized by in silico simulations. Different approaches were developed in an attempt to understand or represent the underlying mechanisms of retention [7,8]. Those are useful when it is necessary to explore a large number of conditions to pick the condition that best fits the requirements. To model the chromatographic behavior of molecules as precisely as possible, several mechanistic models were developed. Some are (semi-)empirical such as the linear solvent strength (LSS) [9] and the Neue–Kuss [10] models. Others are based on the chemical or physical phenomenon or both partitioning such as the linear free energy relationships (LFER) models, the hydrophobic-subtraction model (HSM) [11] or the linear solvation energy relationship (LSER) [12]. They generally offer good performances but they are limited to predicting the retention of compounds that have already been analyzed, therefore they do not completely resolve the issue of the experimental work for new unknown compounds. Several pieces of software enable the performing of these optimizations, among which DryLab™ (Molnar Institute, Berlin, Germany) is possibly the most well-known [13,14]. The development of a method for a new sample of known composition that has never been analyzed before generally requires models using a different approach known as quantitative structure–retention relationship (QSRR) models [15]. The QSRR models are a broad family of models defined only, as its name suggests, by the input and output of the model. They are constructed by establishing a mathematical relationship between structurally derived molecular properties of the compounds that are called molecular descriptors and the target retention time. For this relationship to be assessed, it would need a large number of diversified analytes to cover the range of descriptors for the analytes typically encountered in this analytical context [16]. Unlike other retention theories, multiple generic underlying models were applied. Some examples of implemented models are the multi-linear regression (MLR) [17,18] or the partial least squares (PLS) regression [19,20] but more advanced models such as decision tree-based methods [21,22,23] or artificial neural networks [24,25] were also used.

However, QSRR models are often limited to predictions for the single chromatographic condition used for their training [7]. Different research teams have proposed alternatives to overcome this constraint. Muteki et al., proposed an improved QSRR model with descriptors of the mobile and stationary phases adjoining the descriptors of the compounds with an L-PLS model [26]. Taraji et al., proposed joining the outputs of several QSRR models with RSM [27]. Wiczling et al., proposed a mechanistic multilevel Bayesian model for monoprotic compounds. They concluded that providing experimental data (at least four for mixtures of compounds) was needed to have accurate predictions [28,29].

Joining and optimizing multiple QSRR models with RSM followed by an MCDA would offer a remarkably interesting strategy to assist and accelerate the method development. In this study, a different way of selecting the RSM equation will be tested. The proposed selection will be based on the pKa of the modeled compound to reflect the knowledge of the chemical properties in the retention behavior. The different criteria are calculated in a way to make them robust to the prediction error. A new approach for the MCDA is also developed to work with a distribution of weights instead of fixed values. The proposed strategy allows the user to consider different characteristics desired for the developed method while attributing their relative importance without the complexity of assigning the exact weights that would represent their reasoning. The work presented here is part of a strategy to carry out in silico screening for a reversed-phase liquid chromatography method for small pharmaceutical compounds. In the whole strategy, it would be preceded by QSRR models built on a dataset composed of diverse pharmaceutical-related molecules. Doing this makes it applicable to a wide range of compounds. Those models predict the retention time of known new compounds in known conditions. The objective of this study is to demonstrate that this part of the strategy is efficient and adequate when combined with QSRR models. To demonstrate the good performance of this part of the strategy, it will be applied directly to experimental retention times to assess this part independently and be free from the error of the QSRR models.

## 2. Results and Discussion

This article is divided into two sections focusing on the RSM and the MCDA defined in this study. Each of them will use a specific set of compounds for the different evaluations. For the RSM section, a small test set composed of 4-nitrophenol, ibuprofen, papaverine, and pindolol is used. For the MCDA section, a bigger test set is used to put the strategy under stress. This larger test set is composed of 2,2′-bipyridine, 4-nitrophenol, ibuprofen, metoclopramide, papaverine, pindolol, and verapamil.

### 2.1. Response Surface Methodology

#### 2.1.1. Model Development

All the models were built following the procedure described hereunder. Following the principles of design of experiments for factors of more than two levels (there are five levels for the pH), the response surface methodology is applied for each compound. More precisely, it means that a polynomial equation composed of the different factors is fitted to tune the factor coefficient to model the response. In our case, an equation composed of the pH and the slope of the organic modifier gradient is built to predict the retention time of the compound. A stepwise regression is commonly used as it will remove or add (or both) different factors in the equation in multiple steps to find the “best” model according to some criteria, for example, the Akaike information criterion. The strategy proposed in this study differs from this last point. This strategy defines three equations that are fixed: Equations (1)–(3). Instead of removing or adding factors, the equations are chosen for specific cases. The different cases are defined based on the fact that the behavior of the retention time of a compound as a function of the pH follows a sigmoidal curve and that the pH range covered is limited Using only an equation with the relevant factors will limit the number of parameters to tune and the prediction of non-relevant chromatographic behaviors (e.g., pH independence for neutral compounds). In this case, the models were not fit to find the shape of the relationship between the retention time and the pH, and the gradient time. Here, the shape of the relationship is known for three specific cases and information about the sample is used to choose the best equation.

Considering the pH range covered (from 2.7 to 8.0), three different chromatographic behaviors can be observed. The first one is when the pH does not influence the retention. In this case, the equation does not contain the pH as a factor (Equation (1)). The second one is when the pH influences the retention but only one plateau of the sigmoidal curve is observable. This happens when the relevant pK_a_ is close to one of the limits of the pH range covered. In this case, the equation contains the pH with a first- and second-degree factor (Equation (2)). The third one is when the pH also has an effect on the retention and two plateaus are observable. This happens when the relevant pK_a_ is in the middle of the pH range covered. In this case, the equation contains a first-, a second-, and a third-degree factor for the pH to approximate the sigmoidal curve (Equation (3)).

The gradient time is modeled as a linear effect and is included as a first-degree factor.

For all the equations, it is the logarithm of the retention time that is modeled because it gives better performances than modeling the retention time without transformation [30].

(1) $$\log(t_{R})=\beta_{0}+\beta_{1}\times t_{G}+\epsilon$$

(2) $$\log(t_{R})=\beta_{0}+\beta_{1}\times \mathrm{pH}+\beta_{2}\times t_{G}+\beta_{11}\times {\mathrm{pH}}^{2}+\beta_{12}\times {\mathrm{pH}*t}_{G}+\epsilon$$

(3) $$\log(t_{R})=\beta_{0}+\beta_{1}\times \mathrm{pH}+\beta_{2}\times t_{G}+\beta_{11}\times {\mathrm{pH}}^{2}+\beta_{111}\times {\mathrm{pH}}^{3}+\beta_{12}\times \mathrm{pH}\times t_{G}+\epsilon$$

To account for the error of the models, a Student’s t distribution was used to define the distribution of retention time for each model. The degrees of freedom of the distribution were corresponding to the degrees of freedom of the model. The distribution was then scaled with the standard deviation of the residuals and located to the predicted retention time before being sampled.

#### 2.1.2. Case Study—Test Set 1

**Figure 1:**
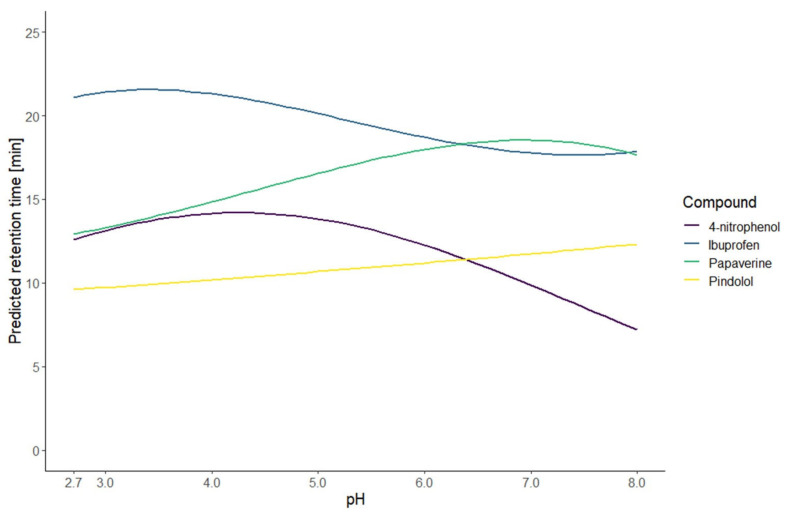
Curves of predicted retention times for the first test set of compounds for all the pH between 2.7 and 8.0 and a gradient time of 20 min.

**Figure 2:**
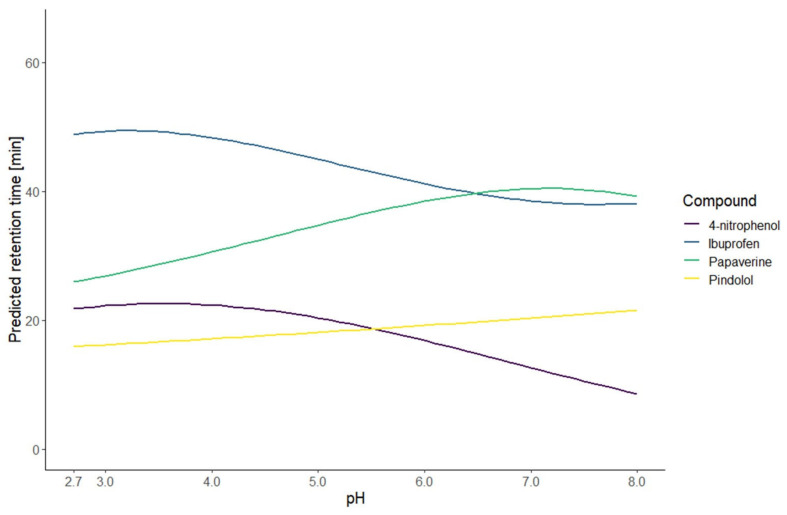
Curves of predicted retention times for the first test set of compounds for all the pH between 2.7 and 8.0 and a gradient time of 60 min.

**Figure 3:**
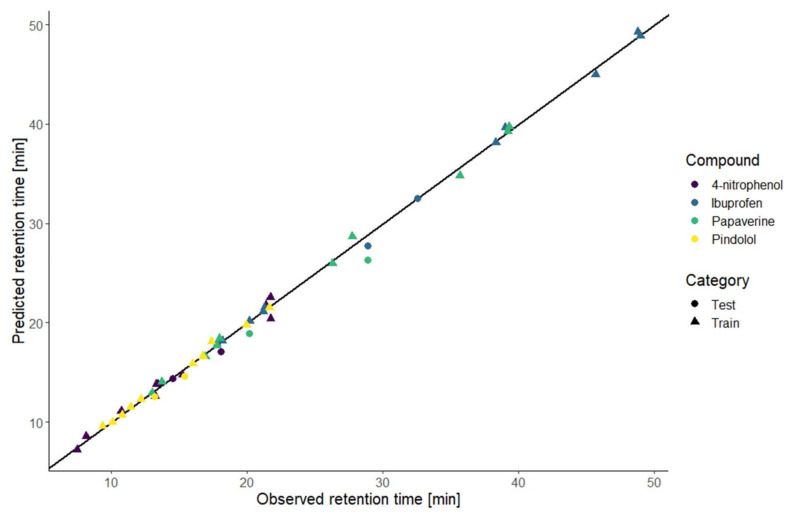
Predicted retention times versus observed retention times of the first test set for the training and the test conditions.

The resulting models of the application of this strategy are illustrated by the predicted retention time curves in Figure 1 and Figure 2. To evaluate the first part of the presented strategy, the models were calculated on the first test set. The performance was evaluated with three measurements: the coefficient of determination (R^2^), the root mean squared error (RMSE), and the mean absolute percentage error (MAPE) of calibration (RMSEC and MAPE_C_) calculated with the data of ten conditions used to build the model and the RMSE and MAPE of prediction (RMSEP and MAPE_P_) calculated with the data of two new external conditions. The values presented in Table 1 show that the model fits the data well. Comparing the values of calibration and prediction, the performance is similar regarding the RMSE, except for the papaverine, meaning that there was no overfitting. The absolute and relative errors shown in Table 2 indicate that the models predicted well the new conditions. For the papaverine, the performances were a bit worse therefore we could expect the prediction to be poor. The comparison of the predicted and observed retention times for the training and new conditions are illustrated in Figure 3. This graphic shows the high correlation between the predicted and observed retention times. Those results indicate that the prediction at an intermediate gradient time does not suffer too much from the limitation to a first-degree factor. Furthermore, the results also demonstrate that the choice of the equation to model the retention time based only on the knowledge of the nature of the compound is enough to have accurate predictions.

### 2.2. Multiple-Criteria Decision Analysis

#### 2.2.1. Method Development

To automate the selection of the best experimental condition, multiple criteria were defined and their responses were gathered into a desirability index.

The following criteria were defined:

- The separation: this was defined as the distance between the peaks of the critical pair at the baseline. It was determined by calculating the difference between the retention times that were sampled from the prediction distributions in each condition for each pair of peaks. From this difference, the half-width of the peaks at the baseline was subtracted to represent the real separation of the peaks. Because only the retention time is predicted, both the left and right expected half-width of each peak must be provided by the user. By allowing to provide a left and a right half-width of the peak, the asymmetry of the peak can be considered. This feature is much more useful in the case of a very large difference in concentration or absorbance of the compounds. One extreme example would be the case of the development of a method for the analysis of the impurities of degradation of an active pharmaceutical ingredient (API). The impurities would be in a much lower concentration than the API hence the peaks are much narrower. Such a difference in peak width is important to consider. For the final calculation of the criteria, the 10% percentile of the distribution of difference for each pair of peaks was conserved and the minimum of those values was kept as the result for each condition (Equation (4));
- The sensitivity to experimental parameters (robustness of the prediction): this was defined as the rate of change of the separation criteria in the function of the experimental parameters. It was determined by calculating the derivative of the separation in the direction of each criterion and then averaging the absolute values of both derivatives (Equation (5));
- The analysis time: this was defined as the minimum analysis time needed to analyze all the compounds in the sample. It was determined by calculating the maximum of the 90% percentile predicted retention time of each compound (Equation (6)).

(4) $$S_{p,g}=\min\left(q_{0.1,i}\left(t_{R,i}-t_{R,i+1}-\left(w_{0,i,l}\times I_{n}\right)-\left(w_{0,i+1,r}\times I_{n}\right)\right)\right),\;i=1,\ldots ,M-1$$

where $S_{p,g}$ is the separation at pH p and gradient time g; $q_{0.1,i}$ is a function that gives the 10% quantile; $t_{R,i}$ is the retention time of compound i when ordered by decreasing retention time; $w_{0,i,l}$ is the left baseline half-width of compound i; $w_{0,i+1,r}$ is the right baseline half-width of compound i + 1; $I_{n}$ is the identity matrix of dimension n; and M is the number of compounds.

(5) $$R_{p,g}=\frac{\left|S_{p}^{\prime }\right|+\left|S_{g}^{\prime }\right|}{2}$$

where $R_{p,g}$ is the sensitivity at pH p and gradient time g; $S_{p}^{\prime }$ is the rate of change of the separation criterion in the direction of the pH for the considered condition; and $S_{g}^{\prime }$ is the rate of change of the separation criterion in the direction of the gradient time for the considered condition.

(6) $$A_{p,g}=\max\left(q_{0.9}\left(t_{R,i}\right)\right),\;i=1,\ldots ,M$$

where $A_{p,g}$ is the analysis time at pH p and gradient time g.

For each of these criteria, a desirability function was defined following the work of Govaerts and Le Bailly de Tilleghem [31]. The desirability function was designed to maximize the separation, maximize the robustness of the prediction, and minimize the analysis time criteria. Each function parameter was calculated from the lower and upper limits of the criterion. For this study, the minimum value of the criteria was used as the lower limit and the maximum value of the criteria as the upper limit. With this definition, the user does not need to specify the different limits for each criterion. The response of each of these desirability functions was then gathered in a single value, the desirability index, by calculating their weighted geometric mean. The geometric mean is used because it is more restrictive. It would cancel out (the desirability index would be zero) in the extreme case the desirability of one criterion is zero [5]. The best condition (with the highest desirability index) represents the best compromise between the different criteria that are available (Equation (7)).

The weights are specified by relative importance that emulates the needs of the user:

- The separation criterion was assigned a weight of 1. This is the main criterion to achieve a “must have”;
- The sensitivity to experimental parameters criterion was assigned a weight of 0.5;
- The analysis time criterion was assigned a weight of 0.1. A short analysis time is preferable but not at the cost of separation. It is “nice to have”.

(7) $$D_{p,g}={\left(d_{S}\left(S_{p,g}\right)\right)}^{w_{S}}\times {\left(d_{R}\left(R_{p,g}\right)\right)}^{w_{R}}\times {\left(d_{A}\left(A_{p,g}\right)\right)}^{w_{A}}$$

where $D_{p,g}$ is the desirability index at pH p and gradient time g; $d_{i}$ is the desirability function for criteria i; and $w_{S}$ is the weight of criteria i.

The desirability index threshold determining the optimal conditions was chosen so that the separation criterion is always greater than 0. In the case of the second test set, this corresponded to a desirability index of 0.5.

To render the desirability index less sensitive to the arbitrary choice of the weights, a Dirichlet distribution was constructed based on the previously defined weights multiplied by a factor representing the confidence in those weights. The different ratios of the weights define the location of the distribution. The confidence factor will define the scale of the distribution and it is set at 100 in this study. From this distribution, multiple vectors of weights were sampled and used to calculate the probability to reach the desirability index threshold. This probability is set at 30% because it was for the screening phase and a high chance of success was not needed.

#### 2.2.2. Case Study—Test Set 2

**Figure 4:**
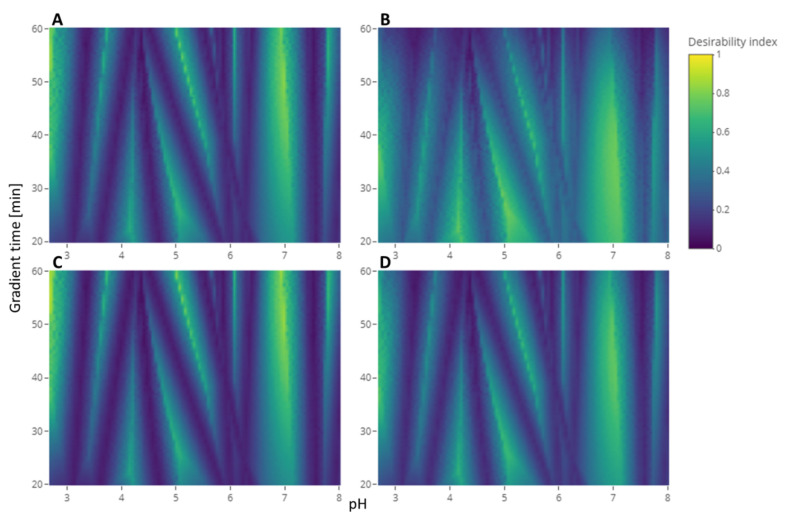
Heatmap of the desirability index in function of the pH and the gradient time calculated with different relative weights for the comparison of the effect of the weights on the desirability index. (**A**): the weights are 1 for the separation criterion, 0.5 for the sensitivity to experimental parameters criterion, and 0.1 for the analysis time criterion. (**B**): the weights are 1, 1 and 1. (**C**): the weights are 1, 0.4, and 0.05. (**D**): the weights are 1, 0.5, and 0.3.

The MCDA strategy was evaluated with the second test set. For the separation criterion, the half-width of all the peaks was defined at 0.5 min. Figure 4 shows different heatmaps of the desirability index of the experimental conditions with different weights attributed to each criterion. Figure 4A shows the heatmap with the weights that we proposed for this strategy: 1 for the separation criterion, 0.5 for the sensitivity to experimental parameters criterion, and 0.1 for the analysis time criterion. It can be compared to the heatmap of the application of the same strategy without weighting the criteria differently shown in Figure 4B where all the weights are 1. Giving the same importance to all the criteria leads to less relative importance of the separation criteria, which results in a slimming of the dark blue bands characteristic of the coelution of two compounds. On the contrary, Figure 4C shows the heatmap where the weights for the sensitivity to the experimental parameters and the analysis time criteria are decreased a little, increasing the relative importance of the separation criterion. On this heatmap, compared to the original, the slim yellow band showing the best condition is brighter. This phenomenon is even more clear in the upper part of the graph because those regions with long gradient times are less penalized by the analysis time criterion. The opposite of this last phenomenon can be observed in Figure 4D, where this part of the graph is darker than the original. This is resulting from the increase in the analysis criterion weight. The comparison of those heatmaps exhibited the sensitivity of the resulting desirability index to the weights assigned by the user to each criterion. Added to the difficulty for the user to find the correct arbitrary value that will represent its interest the best, complementing the calculation of the desirability index with a distribution of weights would give more confidence in the result. Indeed, after selecting the threshold of the desirability index, the probability to reach this threshold was calculated with different combinations of weights sampled from the distribution. The conditions selected as a result would have a high desirability index in a lot of the weights combinations meaning it was not sensitive to the variations of those weights.

**Figure 5:**
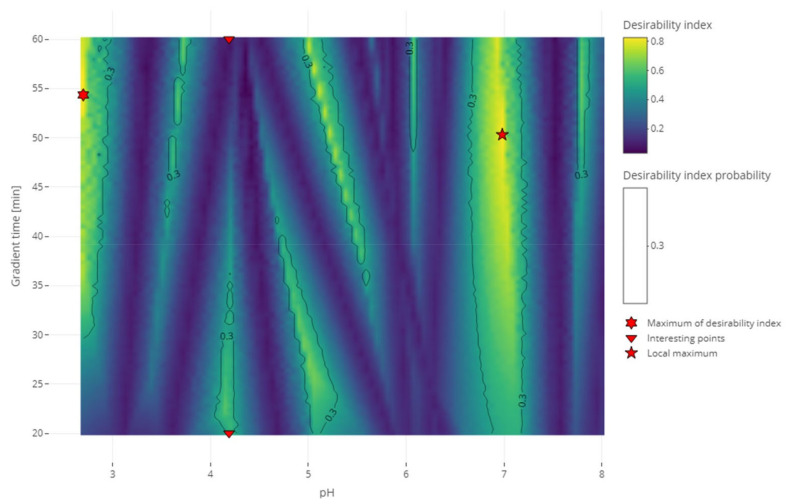
Heatmap of the desirability index in function of the pH and the gradient time calculated with the relative weights of 1 for the separation criterion, 0.5 for the sensitivity to experimental parameters criterion, and 0.1 for the analysis time criterion. The black contour line delimits the regions where there is a probability of 30% or more to reach the desirability index of 0.5. The hexagram shows the condition with the highest desirability index. The five-branch star shows the condition with the highest desirability index inside the largest region. The triangles are interesting points to evaluate the effect of gradient time.

**Figure 6:**
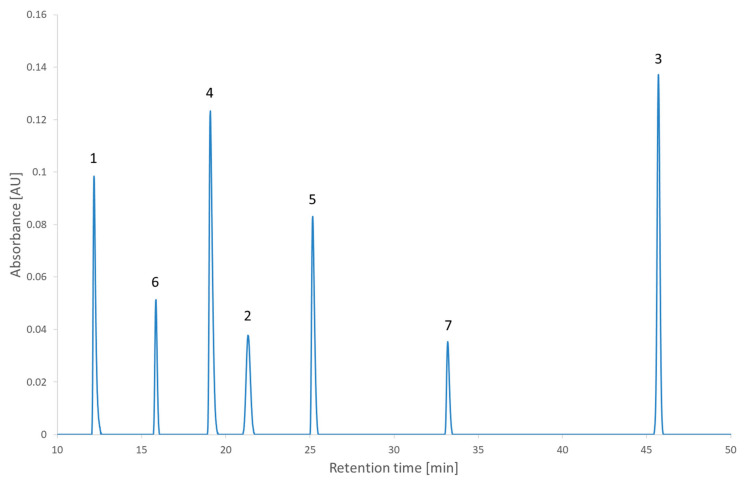
The chromatogram extracted at 210 nm of the second test set analyzed in the condition of pH 2.7 and a gradient time of 54 min. 1: 2,2′-bipyridine, 2: 4-nitrophenol, 3: ibuprofen, 4: metoclopramide, 5: papaverine, 6: pindolol, 7: verapamil.

**Figure 7:**
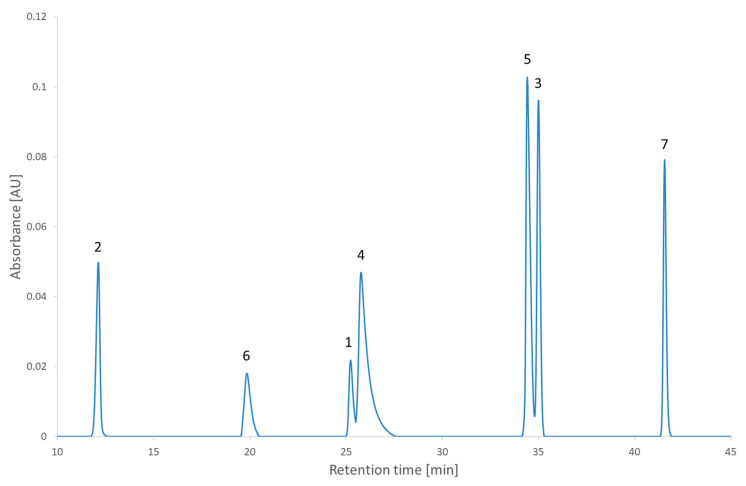
The chromatogram extracted at 210 nm of the second test set analyzed in the condition of pH 7.0 and a gradient time of 50 min. 1: 2,2′-bipyridine, 2: 4-nitrophenol, 3: ibuprofen, 4: metoclopramide, 5: papaverine, 6: pindolol, 7: verapamil.

Figure 5 shows the results of the RSM combined with the MCDA strategy applied to the second test set. One can see that the coelution of different compounds is clearly identified with the dark bands of the desirability index. Different regions with a high desirability index can also be identified. Depending on the objective of the analyst, some of those regions can be selected to continue the method development further. The recommendations regarding the lifecycle management of analytical methods begin to change (the International Council for Harmonisation (ICH) is updating the Q2 and creating the Q14 guidelines) [32,33]. This will impact the way the analytical methods are developed. Instead of only focusing on the separation (the original objective of HPLC) and the robustness (the objective of analytical quality by design), the newly developed method could be developed to attain other objectives in anticipation of its lifecycle. One of the modifications presented in the ICH Q14 under public consultation is the submission of parameter ranges instead of parameter values for the analytical conditions and that, provided it is regulatory approved, moving within the parameter range does not require notifying the regulatory authorities [33]. To give a few examples of when the need to change the analytical conditions occurs in a regulated environment, it could happen for a method used to analyze the impurities during stability studies or used the quality control of a drug product [34]. In the case of the stability studies, the method is developed considering the impurities observed at the beginning of the stability study, but a new impurity might appear and if it coelutes with another of the peaks it would be required to conduct the analysis in another condition. In the case of quality control, a change of condition could be needed after a change of excipient. Although the strategy presented in this article is about the screening phase, it is relevant to already account for the objectives to achieve in the final method. Robustness has already been discussed as being part of the strategy presented in this study thanks to the second criterion. Regarding the lifecycle of the method and the possibility to change the conditions during its lifetime, this is an ambition that will be examined at the end of the strategy. To account for those objectives, two conditions will be investigated. The first one is the condition with the highest desirability index value overall (the global maximum) and the second one is the condition with the highest desirability index value in the largest region selected (local maximum). Those conditions are highlighted in Figure 5 and were realized experimentally to assess the impact of the decision. The experimental chromatogram of the global maximum is illustrated in Figure 6. The resulting separation was particularly good. Although this is only illustrating the screening step, this is the kind of result that would be expected from the optimization step. Figure 7 illustrates the chromatogram of a local maximum. Even though the desirability index was high, the actual peaks were not well separated. Indeed, two pairs of peaks (2,2′-bipyridine and metoclopramide, and ibuprofen and papaverine) coeluted nearly completely. This outcome is explained by the error in the prediction. From Table S4 (Supplementary Materials), it is evident that the two coelutions come from the under-evaluation of the predicted retention time of one of the compounds in each pair. This under evaluation is also observed with the predictions of the global maximum but in that case, the peaks are so distant from each other that it does not impair the actual separation. Supposing the separation of the coeluting peaks could be resolved during the optimization phase, it would be recommended to conduct the analyses corresponding to the highest desirability indices to confirm the selection of the region to continue the method development.

**Figure 8:**
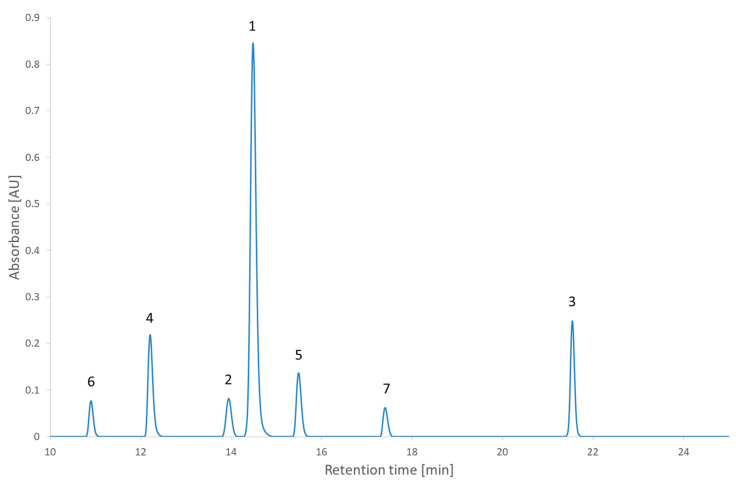
The chromatogram extracted at 210 nm of the second test set analyzed in the condition of pH 4.2 and a gradient time of 20 min. 1: 2,2′-bipyridine, 2: 4-nitrophenol, 3: ibuprofen, 4: metoclopramide, 5: papaverine, 6: pindolol, 7: verapamil.

**Figure 9:**
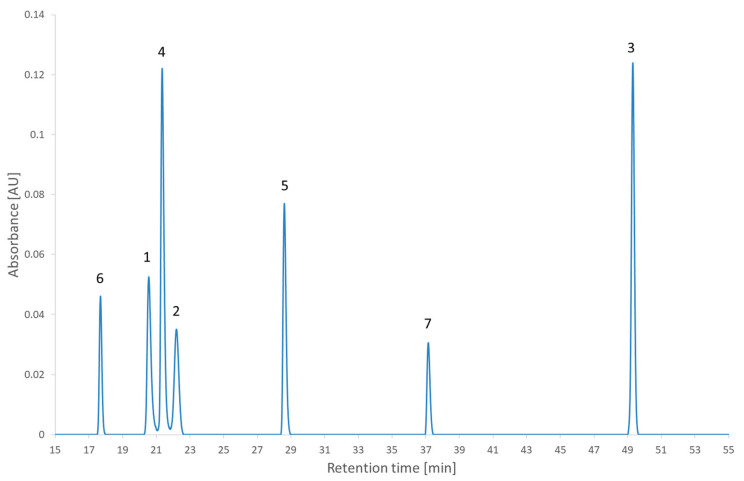
The chromatogram extracted at 210 nm of the second test set analyzed in the condition of pH 4.2 and a gradient time of 60 min. 1: 2,2′-bipyridine, 2: 4-nitrophenol, 3: ibuprofen, 4: metoclopramide, 5: papaverine, 6: pindolol, 7: verapamil.

In Figure 5, two other interesting points are highlighted. They correspond to two conditions at the same pH but with gradient times at the two ends of the range. They were chosen because the condition with the smallest gradient time is considered to be better than the one with the longest retention time. This would usually be considered counterintuitive. From the comparison of Figure 8 and Figure 9, the expected increase in all the retention times can be seen. It could also be observed that the peaks of the chromatogram at a 20 min gradient time were better separated than the peaks of the chromatogram at 60 min. This confirms the results of the RSM and the MCDA. These results can be interpreted by the different influences that the change of gradient time will have on each compound. In this second test set, nearly all the compounds have an increased retention time and stay in the same order. The exception is the 2,2′-bipyridine, which was highly influenced by the gradient time, revealed by its shift of position in the order of elution. These results confirm (if needed) that the gradient time can play a role in the selectivity of an RPLC method [35,36].

## 3. Materials and Methods

### 3.1. Methodology

The part of the strategy presented is structured with the following steps: a response surface model is fitted for each compound in the sample based on experimental retention times. Those retention times replace the retention times predicted by QSRR models that would be used when the complete strategy is applied. To be able to account for the error in the model, a distribution is adapted to the residuals of each model. For a model fitted with the ordinary least squares method, the residuals are expected to follow a normal distribution. Nonetheless, for this study, a more robust alternative, the Student’s t distribution, is used. Its parameters are the mean and the variance of the residuals and the residual degrees of freedom of the model. Predicted retention times in new conditions are sampled from those distributions to calculate different criteria useful for the selection of the best conditions. A desirability function is designed for each of those criteria to rescale them. The output of those desirability functions for all conditions is then aggregated in a single value, the desirability index, which will define the best conditions. This aggregation allows attributing different weights for each criterion. Those weights will be sampled from a Dirichlet distribution which allows for calculating the probability of reaching a specific desirability index while the weights slightly vary.

### 3.2. Chemicals and Reagent

Ammonium bicarbonate, ammonium formate, and formic acid 99% were purchased from VWR Chemicals (Leuven, Belgium). Milli-Q water from a Merck milli-Q pump. Methanol HPLC gradient grade was purchased from J.T. Baker (Deventer, Netherlands).

Standard compounds: 2,2′-bipyridine, 4-nitrophenol, ibuprofen, metoclopramide, papaverine, verapamil hydrochloride from TCI; pindolol from Abcam (Rozenburg, Netherlands).

### 3.3. Instrumentation and Chromatographic Conditions

The following compounds were selected for the application of the strategy presented in this study for their diverse chromatographic behaviors: 2,2′-bipyridine, 4-nitrophenol, ibuprofen, metoclopramide, papaverine, pindolol, and verapamil.

The strategy is designed to be applied to predicted retention times in different conditions. Those predicted retention times are replaced by experimental retention times to evaluate the performance of the strategy. The experimental retention times are taken from the dataset described in extenso elsewhere [37]. The complete dataset is composed of ninety-eight compounds analyzed in reversed-phase liquid chromatography at five pH values (2.7, 3.5, 5.0, 6.5, and 8.0) and two gradient times (0% to 95% methanol in 20 and 60 min) on two C18 columns Waters XSelect HSS T3 2.1 × 100 mm 3.5 μm on three different chromatography systems.

New conditions were realized experimentally to control the prediction performance of new conditions. The samples were analyzed on a Waters Alliance 2695 HPLC coupled with a UV-visible photodiode array detector 2996 module. The separation was achieved on a C18 stationary phase Waters XSelect HSS T3 2.1 × 100 mm 3.5 μm and the flow rate was fixed at 0.3 mL/min. The analytical method was a gradient from 0% to 95% methanol in 40 min followed by a 5 min hold. Different buffers were used to set the pH (3.0 and 6.0) of the mobile phase for the new conditions. The injection volume was set at 10 μL, the column temperature was set at 25°C and the PDA was set to acquire from 209 to 395 nm.

### 3.4. Sample Preparation

The stock solutions were prepared in water, methanol, or a mixture of both depending on the solubility properties of each compound. The subsequent single compound solutions and mixture solutions were prepared by diluting the stock solutions using water or a mixture of water and methanol to reach the target concentration of 20 μg·mL^−1^. A more concentrated solution (40 μg·mL^−1^) was necessary to detect the ibuprofen. For detailed information about each compound preparation, see Table S2 in Supplementary Materials.

Two test sets were used to assess the strategy. The first one was composed of 4-nitrophenol, ibuprofen, papaverine, and pindolol. The second one was composed of 2,2′-bipyridine, 4-nitrophenol, ibuprofen, metoclopramide, papaverine, pindolol, and verapamil.

### 3.5. Software

Waters^®^ Empower 3 Workgroup (Waters, Milford, MA, USA) was used to control the chromatographic system and to acquire and manage the data.

R version 4.1.1, the packages MCMCpack version 1.6-0, igraph version 1.2.7, and raster version 3.5-2 were used for the analysis of the results.

## 4. Conclusions

A strategy combining RSM modeling of the retention time of known compounds based on their chemical properties and MCDA robust to the weights attributed by the user was designed with the intention of offering the opportunity to perform an in silico screening as a first step in the method development of a known sample when associated with QSRR models. The presented results showed that the developed strategy could help with the screening phase of the method development. The strategy offers the possibility of reducing the range of the parameters for the optimization step by considering criteria commonly used while taking decisions during method development.

One of the shortcomings of the strategy is the use of a Student’s t distribution after the models to propagate the error. The use of Bayesian models instead would be a better alternative. First, it would offer the possibility to provide more information through the prior distribution of parameters. Second, it would offer the opportunity to directly have access to this error of the model from the ensuing posterior distribution.

One of the limitations of the strategy is that the user needs to provide the expected width of the peaks. To overcome this limitation, one solution could be to define them as proportional to the ratio of the concentration. Another solution would be to make a first single injection to collect the real width. However, this limitation is minor because this is only for the screening step and would be followed by the optimization step.

The advantages of this strategy are that it limits the sensitivity of the decision to the weights allocated at the different criteria. Furthermore, thanks to the different criteria implemented, the strategy allows the user to consider multiple criteria in line with its objectives from the beginning of the method development. After the recommended experimental verification, the selected method would continue to be developed in a region where the desirability index would vary moderately, granting the possibility to adapt the condition without needing to start the whole method development from the beginning.

A long-term objective of the complete strategy would be to develop a user interface to help and guide the user providing all the required information (peak width, weights corresponding to the output of the development, confidence in the weights, desirability index threshold, and probability to reach the desirability index).

## Figures and Tables

**Table 1:** Individual and global performance of models in calibration. tR is the retention time at pH 5.0 with a gradient time of 20 min.

Compound	t_R_ [min]	R^2^	RMSEC [min]	MAPEC [%]	RMSEP [min]	MAPEP [%]
**4-nitrophenol**	13.35	0.984	0.65	3.91	0.73	3.64
**Ibuprofen**	20.22	0.999	0.35	0.75	0.83	2.25
**Papaverine**	16.97	0.998	0.48	1.61	2.06	8.38
**Pindolol**	10.83	0.996	0.27	1.39	0.75	5.47

**Table 2:** Absolute and relative errors of the compound of the first test set in the two new conditions. Condition 1: pH 3.0 and 40 min gradient time. Condition 2: pH 6.0 and 40 min of gradient time.

Compound	Condition 1		Condition 2	
	Absolute Error [min]	Relative Error [%]	Absolute Error [min]	Relative Error [%]
**4-nitrophenol**	−0.82	−4.50	−0.16	−1.12
**Ibuprofen**	−0.10	−0.30	−0.88	−3.03
**Papaverine**	−1.07	−5.28	−2.00	−6.92
**Pindolol**	−0.56	−4.24	−0.65	−4.18

## Data Availability

The data set presented in this study is openly available in Mendeley Data at doi: 10.17632/csm5gsmr5t.1, doi: 10.17632/2w64h8pvkc.1 and doi: 10.17632/7v5p4gsh4z.1. The data of the new conditions used for the external validation are available in the Supplementary Data.

## Supplementary Materials

The following supporting information can be downloaded at: https://www.mdpi.com/article/10.3390/molecules27238306/s1, Table S1: Solubilization and dilution solutions of standard compounds, Table S2: Predicted and observed retention times of the second test set of compounds in the condition with pH 2.7 and 54 min gradient time, Table S3: Predicted and observed retention times of the second test set of compounds in the condition with pH 7.0 and 50 min gradient time, Table S4: Retention times of the compounds of the test set 1 in the new conditions and the condition parameters.

## References

[1] Vera Candioti L., de Zan M.M., Cámara M.S., Goicoechea H.C. (2014). Experimental Design and Multiple Response Optimization. Using the Desirability Function in Analytical Methods Development. Talanta.

[2] Czitrom V. (1999). One-Factor-at-a-Time versus Designed Experiments. Am. Stat..

[3] Hibbert D.B. (2012). Experimental Design in Chromatography: A Tutorial Review. J. Chromatogr. B Anal. Technol. Biomed. Life Sci..

[4] Bezerra M.A., Santelli R.E., Oliveira E.P., Villar L.S., Escaleira L.A. (2008). Response Surface Methodology (RSM) as a Tool for Optimization in Analytical Chemistry. Talanta.

[5] Bezerra M.A., Ferreira S.L.C., Novaes C.G., dos Santos A.M.P., Valasques G.S., da Mata Cerqueira U.M.F., dos Santos Alves J.P. (2019). Simultaneous Optimization of Multiple Responses and Its Application in Analytical Chemistry—A Review. Talanta.

[6] Costa N.R., Lourenço J., Pereira Z.L. (2011). Desirability Function Approach: A Review and Performance Evaluation in Adverse Conditions. Chemom. Intell. Lab. Syst..

[7] Haddad P.R., Taraji M., Szücs R. (2021). Prediction of Analyte Retention Time in Liquid Chromatography. Anal. Chem..

[8] Den Uijl M.J., Schoenmakers P.J., Pirok B.W.J., van Bommel M.R. (2021). Recent Applications of Retention Modelling in Liquid Chromatography. J. Sep. Sci..

[9] Snyder L.R., Dolan J.W., Gant J.R. (1979). Gradient Elution in High-Performance Liquid Chromatography. I. Theoretical Basis for Reversed-Phase Systems. J. Chromatogr. A.

[10] Neue U.D., Kuss H.J. (2010). Improved Reversed-Phase Gradient Retention Modeling. J. Chromatogr. A.

[11] Snyder L., Dolan J., Carr P. (2004). The Hydrophobic-Subtraction Model of Reversed-Phase Column Selectivity. J. Chromatogr. A.

[12] Sadek P.C., Carr P.W., Doherty R.M., Kamlet M.J., Taft R.W., Abraham M.H. (1985). Study of Retention Processes in Reversed-Phase High-Performance Liquid Chromatography by the Use of the Solvatochromic Comparison Method. Anal. Chem..

[13] Fekete S., Murisier A., Nguyen J.M., Lauber M.A., Guillarme D. (2021). Negative Gradient Slope Methods to Improve the Separation of Closely Eluting Proteins. J. Chromatogr. A.

[14] Ferencz E., Kelemen É.-K., Obreja M., Sipos E., Vida S., Urkon M., Szabó Z.-I. (2021). Computer-Assisted UHPLC Method Development and Optimization for the Determination of Albendazole and Its Related Substances. J. Pharm. Biomed. Anal..

[15] Héberger K. (2007). Quantitative Structure-(Chromatographic) Retention Relationships.

[16] Kaliszan R. (2007). QSRR: Quantitative Structure-(Chromatographic) Retention Relationships. Chem. Rev..

[17] Baczek T., Wiczling P., Marszałł M., vander Heyden Y., Kaliszan R. (2005). Prediction of Peptide Retention at Different HPLC Conditions from Multiple Linear Regression Models. J. Proteome Res..

[18] Goodarzi M., Jensen R., vander Heyden Y. (2012). QSRR Modeling for Diverse Drugs Using Different Feature Selection Methods Coupled with Linear and Nonlinear Regressions. J. Chromatogr. B Analyt. Technol. Biomed. Life Sci..

[19] Randazzo G.M., Tonoli D., Hambye S., Guillarme D., Jeanneret F., Nurisso A., Goracci L., Boccard J., Rudaz S. (2016). Prediction of Retention Time in Reversed-Phase Liquid Chromatography as a Tool for Steroid Identification. Anal. Chim. Acta.

[20] Bodzioch K., Durand A., Kaliszan R., Baczek T., vander Heyden Y. (2010). Advanced QSRR Modeling of Peptides Behavior in RPLC. Talanta.

[21] Zheng L., Watson D.G., Johnston B.F., Clark R.L., Edrada-Ebel R., Elseheri W. (2009). A Chemometric Study of Chromatograms of Tea Extracts by Correlation Optimization Warping in Conjunction with PCA, Support Vector Machines and Random Forest Data Modeling. Anal. Chim. Acta.

[22] Cao M., Fraser K., Huege J., Featonby T., Rasmussen S., Jones C. (2015). Predicting Retention Time in Hydrophilic Interaction Liquid Chromatography Mass Spectrometry and Its Use for Peak Annotation in Metabolomics. Metabolomics.

[23] Naylor B.C., Leon Catrow J., Alan Maschek J., Cox J.E. (2020). QSRR Automator: A Tool for Automating Retention Time Prediction in Lipidomics and Metabolomics. Metabolites.

[24] Petritis K., Kangas L.J., Ferguson P.L., Anderson G.A., Paša-Tolić L., Lipton M.S., Auberry K.J., Strittmatter E.F., Shen Y., Zhao R. (2003). Use of Artificial Neural Networks for the Accurate Prediction of Peptide Liquid Chromatography Elution Times in Proteome Analyses. Anal. Chem..

[25] Kaliszan R., Baczek T., Bucinḱnski A., Buszewski B., Sztupecka M. (2003). Prediction of Gradient Retention from the Linear Solvent Strength (LSS) Model, Quantitative Structure-Retention Relationships (QSRR), and Artificial Neural Networks (ANN). J. Sep. Sci..

[26] Muteki K., Morgado J.E., Reid G.L., Wang J., Xue G., Riley F.W., Harwood J.W., Fortin D.T., Miller I.J. (2013). Quantitative Structure Retention Relationship Models in an Analytical Quality by Design Framework: Simultaneously Accounting for Compound Properties, Mobile-Phase Conditions, and Stationary-Phase Properties. Ind. Eng. Chem. Res..

[27] Taraji M., Haddad P.R., Amos R.I.J., Talebi M., Szucs R., Dolan J.W., Pohl C.A. (2017). Rapid Method Development in Hydrophilic Interaction Liquid Chromatography for Pharmaceutical Analysis Using a Combination of Quantitative Structure-Retention Relationships and Design of Experiments. Anal. Chem..

[28] Wiczling P., Kubik Ł., Kaliszan R. (2015). Maximum A Posteriori Bayesian Estimation of Chromatographic Parameters by Limited Number of Experiments. Anal. Chem..

[29] Wiczling P., Kaliszan R. (2016). How Much Can We Learn from a Single Chromatographic Experiment? A Bayesian Perspective. Anal. Chem..

[30] Lebrun P., Govaerts B., Debrus B., Ceccato A., Caliaro G., Hubert P., Boulanger B. (2008). Development of a New Predictive Modelling Technique to Find with Confidence Equivalence Zone and Design Space of Chromatographic Analytical Methods. Chemom. Intell. Lab. Syst..

[31] Govaerts B., le Bailly de Tilleghem C. (2005). Distribution of Desirability Index in Multicriteria Optimization Using Desirability Functions Based on the Cumulative Distribution Function of the Standard Normal.

[32] International Conference on Harmonization Validation of Analytical Procedures Q2(R2). https://www.ich.org/page/quality-guidelines.

[33] International Conference on Harmonization Analytical Procedure Development Q14 (Draft Version). https://www.ich.org/page/quality-guidelines.

[34] Hubert C., Lebrun P., Houari S., Ziemons E., Rozet E., Hubert P. (2014). Improvement of a Stability-Indicating Method by Quality-by-Design versus Quality-by-Testing: A Case of a Learning Process. J. Pharm. Biomed. Anal..

[35] Tyteca E., Vanderlinden K., Favier M., Clicq D., Cabooter D., Desmet G. (2014). Enhanced Selectivity and Search Speed for Method Development Using One-Segment-per-Component Optimization Strategies. J. Chromatogr. A.

[36] (2017). Watson Dawn Wallace The Secrets of Successful Gradient Elution. LC-GC N. Am..

[37] Van Laethem T., Kumari P., Hubert P., Fillet M., Sacré P.-Y., Hubert C. (2022). A Pharmaceutical-Related Molecules Dataset for Reversed-Phase Chromatography Retention Time Prediction Built on Combining PH and Gradient Time Conditions. Data Brief.

