# Supplementary material for: User-Driven Strategy for In Silico Screening of Reversed-Phase Liquid Chromatography Conditions for Known Pharmaceutical-Related Small Molecules
Source: Molecules. 2022 Nov 28;27(23):8306. doi: 10.3390/molecules27238306 (PMC9735675; doi:10.3390/molecules27238306)
Supplement: Supplementary file 1 [file molecules-27-08306-s001.zip › molecules-1974087-supplementary.pdf]

Supplementary materials of "User-Driven Strategy for In Silico Screening of Reversed-Phase Liquid Chromatography Conditions for Known Pharmaceutical-Related Small Molecules"

Thomas Van Laethem [1,2,*], Priyanka Kumari [1,2], Bruno Boulanger [3], Philippe Hubert [2], Marianne Fillet [1], Pierre-Yves Sacré [2] and Cédric Hubert [2,*]

[1] Laboratory for the Analysis of Medicines, University of Liège (ULiège), CIRM, B36 Tower 4 (route 688
CHU) +3, Avenue Hippocrate, 15, 4000 Liège, Belgium

[2] Laboratory of Pharmaceutical Analytical Chemistry, University of Liège (ULiège), CIRM, B36 Tower 4 (route 688 CHU) +2, Avenue Hippocrate, 15, 4000 Liège, Belgium

[3] Pharmalex Belgium, Rue Edouard Belin, 5, 1435 Mont-Saint-Guibert, Belgium

* Correspondence: tvanlaethem@uliege.be (T.V.L.); chubert@uliege.be (C.H.)

Table S1. Solubilization and dilution solutions of standard compounds.

Compound	Solubilization solution	Dilution solution	Initial concentration [$mg.ml^{-1}$]	Final concentration [$mg.ml^{-1}$]
**2,2'-Bipyridine**	MeOH	H_2O	1	0.02
**4-Nitrophenol**	MeOH	H_2O	1	0.02
**Ibuprofen**	MeOH	H_2O	1	0.04
**Metoclopramide**	MeOH	H_2O	1	0.02
**Papaverine**	MeOH	H_2O	1	0.02
**Pindolol**	H_2O/MeOH 50:50	H_2O	0.5	0.02
**Verapamil**	H_2O	H_2O	1	0.02

Table S2. Predicted and observed retention times of the second test set of compounds in the condition with pH 2.7 and 54 minutes gradient time.

Number	Compound	Predicted t_R [min]	Observed t_R [min]
**1**	2,2′-bipyridine	12.35	12.17
**6**	pindolol	14.94	15.95
**4**	metoclopramide	18.02	19.19
**2**	4-nitrophenol	20.42	21.35
**5**	papaverine	23.91	25.28
**7**	verapamil	30.91	33.21
**3**	ibuprofen	43.42	45.79

Table S3. Predicted and observed retention times of the second test set of compounds in the condition with pH 7.0 and 50 minutes gradient time.

Number	Compound	Predicted t_R [min]	Observed t_R [min]
**2**	4-nitrophenol	11.97	12.13
**6**	pindolol	17.89	19.86
**1**	2,2′-bipyridine	24.96	25.24
**4**	metoclopramide	22.86	25.78
**3**	ibuprofen	32.27	34.48
**5**	papaverine	34.14	34.91
**7**	verapamil	37.50	41.60

Table S4. Retention times of the compounds of the test set 1 in the new conditions and the condition parameters.

Compound	Observed t_R [min]	Real pH	Target pH	Gradient time [min]
**4-Nitrophenol**	18.10	3	3	40
**4-Nitrophenol**	14.56	6.01	6	40
**Ibuprofen**	32.59	3	3	40
**Ibuprofen**	28.93	6.01	6	40
**Papaverine**	20.20	3	3	40
**Papaverine**	28.91	6.01	6	40
**Pindolol**	13.23	3	3	40
**Pindolol**	15.45	6.01	6	40
